# Pharmacokinetics of meropenem during continuous renal replacement therapy in critically ill patients

**DOI:** 10.1186/cc13593

**Published:** 2014-03-17

**Authors:** J Solé Violan, J Ferrer Agüero, B Sádaba, S Sancho, R Zaragoza, P Luque, M Nieto, M López, F García, C Hernández, J Azanza

**Affiliations:** 1 HUGC Dr Negrín, Las Palmas de GC, Spain; 2 Universidad de Navarra, Pamplona, Spain; 3 Hospital Dr Peset, Valencia, Spain; 4 Hospital Clínico, Zaragoza, Spain; 5 Hospital General Yagüe, Burgos, Spain; 6 Hospital Morales Meseguer, Murcia, Spain

## Introduction

Antibiotic dosing for patients with acute renal failure receiving continuous renal replacement therapy (CRRT) is a clinical challenge. The aim of this study was to investigate the pharmacokinetics of meropenem (M) during CRRT.

## Methods

A prospective and multicenter study was conducted at seven hospitals. Fifteen critically ill patients undergoing either continuous venovenous hemofiltration (CVVHF) or hemodiafiltration (CVVHDF) were included. Serum and ultrafiltrate (UF) levels of M were determined by liquid chromatography. Blood samples were drawn 24 hours after starting CRRT at 08:00 a.m., 09:00 a.m., 10:00 a.m., 01:00 p.m., 06:00 p.m., 20:00 p.m. and 08:00 a.m. of the following day. CRRT clearance (Cl), total amount of M in the UF (MUF), percentage of the dose extracted by CRRT (EF) and the AUC (ng/hour/ml) were calculated.

## Results

Nine patients were treated with CVVHDF and six with CVVHF. M (0.5 to 2 g) was administered every 6 to 12 h by i.v. infusion over 15 minutes. Data (mean and SD) concerning the dialysate flow rate (DF; ml/hour), blood flow rate (ml/minute) and the average UF rate (ml/ kg/hour) for CRRT techniques are shown in Table 1. Pharmacokinetic parameters for M are depicted in Table 2. No differences in either EF or M Cl between the two CRRT techniques were observed.

**Table 1:** Parameters of CRRT techniques

	CVVHF (*n *= 6)	CVVHDF (*n *= 9)
DF rate		964/94
Blood flow rate	190/33	187/29
UF rate	37.7/5.7	29.8/15.6

**Table 2:** Pharmacokinetic parameters for meropenem

	CVVHF	CVVHDF
AUC	357/188	433/369
M dose (g/day)	1.7/0.4	2.2/1.4
MUF (mg)	338/104	625/499
EF (%)	28/13	30/26
M Cl (l/hour)	1.2/0.7	1.7/1.5

## Conclusion

A significant removal of M by CVVHF/CVVHDF was observed. Dose adjustment is necessary in critically ill patients receiving CRRT.

